# Gut Microbiota at the Crossroad of Hepatic Oxidative Stress and MASLD

**DOI:** 10.3390/antiox14010056

**Published:** 2025-01-06

**Authors:** Fabrizio Termite, Sebastiano Archilei, Francesca D’Ambrosio, Lucrezia Petrucci, Nicholas Viceconti, Roberta Iaccarino, Antonio Liguori, Antonio Gasbarrini, Luca Miele

**Affiliations:** CEMAD Digestive Diseases Center, Fondazione Policlinico Universitario “A. Gemelli” IRCCS, Università Cattolica del Sacro Cuore, Largo A. Gemelli 8, 00168 Rome, Italysebastiano.archilei01@icatt.it (S.A.);

**Keywords:** metabolic dysfunction-associated steatotic liver disease, gut microbiota, oxidative stress, lipid peroxidation, intestinal permeability, farnesoid X receptor, ferroptosis, trimethylamine N-oxide, platelet activation

## Abstract

Metabolic dysfunction-associated steatotic liver disease (MASLD) is a prevalent chronic liver condition marked by excessive lipid accumulation in hepatic tissue. This disorder can lead to a range of pathological outcomes, including metabolic dysfunction-associated steatohepatitis (MASH) and cirrhosis. Despite extensive research, the molecular mechanisms driving MASLD initiation and progression remain incompletely understood. Oxidative stress and lipid peroxidation are pivotal in the “multiple parallel hit model”, contributing to hepatic cell death and tissue damage. Gut microbiota plays a substantial role in modulating hepatic oxidative stress through multiple pathways: impairing the intestinal barrier, which results in bacterial translocation and chronic hepatic inflammation; modifying bile acid structure, which impacts signaling cascades involved in lipidic metabolism; influencing hepatocytes’ ferroptosis, a form of programmed cell death; regulating trimethylamine N-oxide (TMAO) metabolism; and activating platelet function, both recently identified as pathogenetic factors in MASH progression. Moreover, various exogenous factors impact gut microbiota and its involvement in MASLD-related oxidative stress, such as air pollution, physical activity, cigarette smoke, alcohol, and dietary patterns. This manuscript aims to provide a state-of-the-art overview focused on the intricate interplay between gut microbiota, lipid peroxidation, and MASLD pathogenesis, offering insights into potential strategies to prevent disease progression and its associated complications.

## 1. Introduction

Metabolic dysfunction-associated steatotic liver disease (MASLD), formerly termed non-alcoholic fatty liver disease (NAFLD), is a condition characterized by fat accumulation in the liver in the presence of at least one cardiometabolic risk factor, such as obesity, hypertension, impaired glucose tolerance, or dyslipidemia [1]. MASLD has become the leading cause of chronic liver disease worldwide, impacting over one-third of the adult population and representing the second cause for liver transplantation in the USA [2,3,4]. MASLD includes a broad range of histological stages: simple fat accumulation in the liver (steatosis), which can advance to metabolic dysfunction-associated steatohepatitis (MASH), previously known as non-alcoholic steatohepatitis (NASH), characterized by lobular inflammation and hepatocellular ballooning, fibrosis, and ultimately cirrhosis, which increases the risk of hepatocellular carcinoma [5]. Approximately 15% to 30% of individuals with simple steatosis progress to MASH. While fewer than 5% of simple steatosis advance to cirrhosis, MASH and advanced liver fibrosis, respectively, progress to cirrhosis in 10–15% and 25–30% of patients over a decade [6,7]. Individuals with simple steatosis have a life expectancy that aligns closely with that of the general population [8]. Conversely, patients with MASH face a reduced life expectancy, primarily due to a higher risk of liver-related and cardiovascular complications [9]. 

Recent research has provided new insights into the pathogenesis of MASLD, moving away from the traditional “two-hit” framework towards a model that incorporates “multiple parallel hits”, pathogenic factors, such as insulin resistance, diet, epigenetics, and genetics, as well as gut microbiota, that may synergistically contribute to the progression of simple steatosis to MASH [10]. Oxidative stress represents the crucial effector through which all the aforementioned pathogenic factors converge, leading to hepatocellular death and tissue damage [10].

The gut microbiota is the complex community of microorganisms, including bacteria, fungi, viruses, and other microbes, that reside in the human digestive tract. It is primarily dominated by bacteria from four main phyla (Firmicutes, Bacteroides, Actinobacteria, and Proteobacteria), further subdivided in taxonomic levels: classes, orders, families, genera, and species [11]. In particular, the role of the “gut-liver axis” has become increasingly significant in liver diseases, as alterations in the gut microbiota can directly influence hepatic oxidative stress through various pathogenetic pathways. 

**Figure 1:**
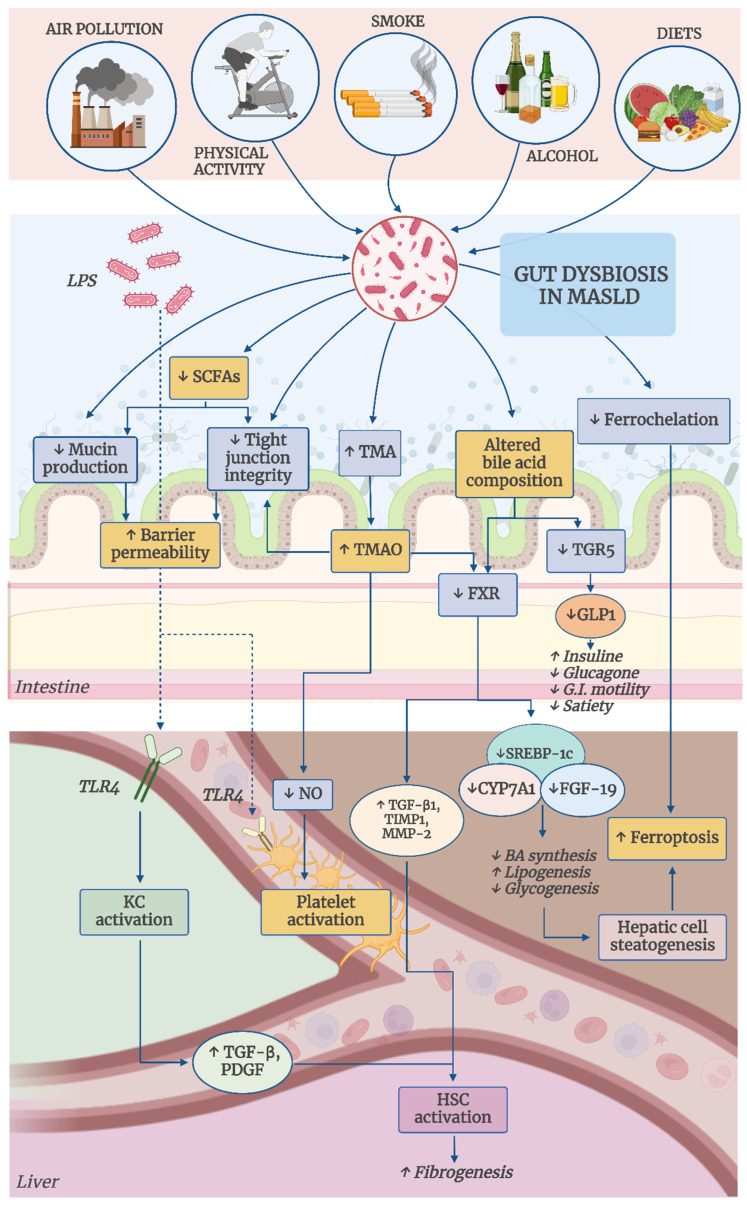
Gut dysbiosis at the crossroad of hepatic oxidative stress and MASLD. BA: bile acids; CYP: cytocrome; FGF-19: fibroblast growth factor 19; FXR: farnesoid X receptor; G.I.: gastro-intestinal; GLP1: glucagon-like peptide-1; HSC: hepatic stellate cell; KC: kuppfer cell; LPS: lipopolysaccharide; MASLD: metabolic dysfunction-associated steatotic liver disease; MMP-2: matrix metalloproteinase 2; NO: nitric oxide; PDGF: platelet-derived growth factor; SCFAs: short-chain fatty acids; SREBP-1c: sterol regulatory element-binding protein 1c; TGF-β: transforming growth factor beta; TGR5: G-protein-coupled bile acid receptor; TIMP1: tissue inhibitor of metalloproteinase 1; TLR4: toll-like receptor 4; TMA: trimethylamine; TMAO: trimethylamine N-oxide.

This manuscript provides an overview of the various pathogenic mechanisms by which an unhealthy microbiota promotes hepatic oxidative stress in MASLD. It also examines the impact of key MASLD risk factors—both environmental and lifestyle-related—on hepatic oxidative stress through alteration of individual microbiota composition. Figure 1 offers a graphical summary of the manuscript. 

## 2. Role of Oxidative Stress in the Pathogenesis of MASLD

Recent studies have provided new insights into the pathogenesis of MASLD, moving beyond the traditional “two-hit” model [12] toward a “multiple parallel hits” hypothesis [13]. This model includes factors like insulin resistance, diet, endotoxemia, genetics, and epigenetics that collectively contribute to the progression from simple steatosis to MASH and fibrosis. Oxidative stress is the key effector through which all the aforementioned pathogenic factors converge, leading to hepatocellular death and tissue damage [14].

Hepatic lipid overload, particularly by free fatty acids (FFAs), triggers excessive oxidant production through various reactive oxygen species (ROS)-generating mechanisms, including superoxide anion (O_2_^−^) and hydroxyl radicals (OH^−^), highly unstable species with unpaired electrons capable of initiating oxidation and generating additional ROS, such as hydrogen peroxide (H_2_O_2_), peroxynitrite (ONOO^−^), and hypochlorous acid (HOCl) [15,16].

**Figure 2:**
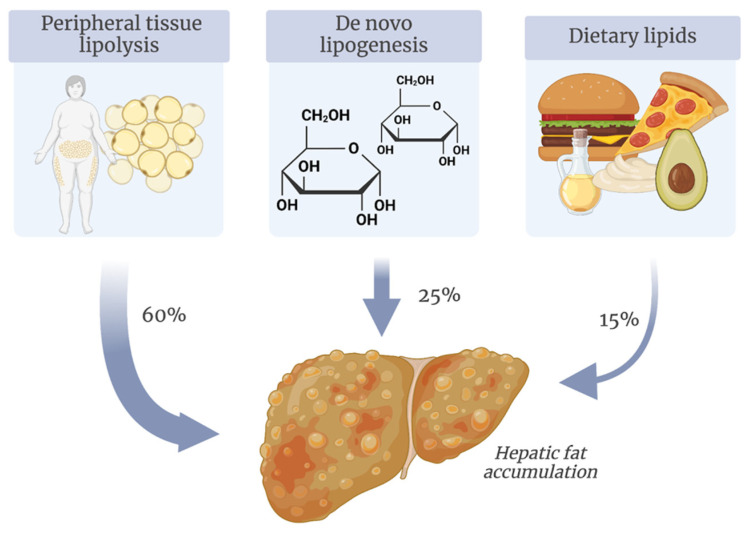
Biochemical sources of hepatic fat accumulation.

More than 50% of hepatic FFAs are derived from peripheral tissue lipolysis, driven by insulin’s effects on adipose tissue [17]. Insulin resistance plays a central role in MASLD pathogenesis, as it increases peripheral lipolysis and FFAs delivery to the liver [18]. The second largest source of hepatic FFAs is de novo lipogenesis, which consists of the conversion of excess dietary glucose into fatty acids [19]. In skeletal muscle insulin resistance, hyperglycemia and hyperinsulinemia activate carbohydrate-response element-binding protein (ChREBP) and sterol regulatory element-binding protein 1c (SREBP1c) in hepatocytes, enhancing lipogenic enzyme expression and hepatic FFAs synthesis from glucose [20]. Dietary lipids represent the third major source of FFAs, whose digestion and absorption are mediated by bile acids and specific bile acid receptors, like farnesoid X receptor (FXR) [21]. Biochemical sources of hepatic fat accumulation are schematically represented in Figure 2. 

Excessive hepatic FFAs accumulation can be managed either by esterification into triglycerides and incorporation into VLDL, a process dependent on choline metabolism, or via mitochondrial beta-oxidation [22]. When the balance between storage and disposal pathways shifts in favor of storage, hepatocytes compensate by maximizing beta-oxidation. FFAs are converted to Acyl-CoA in the cytoplasm, entering mitochondria for beta-oxidation, producing nicotinamide adenine dinucleotide (NADH) and flavin adenine dinucleotide (FADH2). However, this overwhelms the electron transport chain (ETC), leading to electron leakage and ROS production. Acyl-CoA that escapes beta-oxidation enters the tricarboxylic acid (TCA) cycle, generating substrates for gluconeogenesis, thus perpetuating a vicious cycle of “lipid overload-ROS generation” [23]. The production of ROS is counteracted by the action of antioxidants. The antioxidant defense mechanisms consist of both enzymatic and non-enzymatic elements. The enzymatic components include a variety of enzymes that neutralize ROS, such as alpha-dioxygenase (DOX), ascorbate peroxidase (APX), catalase (CAT), glutathione peroxidase (GPX), glutathione reductase (GR), and superoxide dismutase (SOD) [24]. Conversely, the non-enzymatic components are made up of small molecules like glutathione, retinol (vitamin A), ascorbic acid (vitamin C), and tocopherol (vitamin E), which act as electron donors, shielding cellular structures and biomolecules from ROS-induced harm [24].

ROS accumulation damages hepatocyte biomolecules, particularly cardiolipin, a phospholipid within the inner mitochondrial membrane, where the ETC resides [14]. Cardiolipin breakdown disrupts ETC complex function and induces mitochondrial permeability transition (MPT) pore opening, releasing cytochrome C into the cytosol, triggering apoptosis through the inflammasome-caspase-9 pathway [25]. ROS also oxidizes phospholipid membrane polyunsaturated fatty acids (PUFAs), initiating a chain reaction of lipid peroxidation catalyzed by ferrous iron (Fe^2+^), a process known as ferroptosis [26]. Ferroptosis has recently emerged as a key pathogenic mechanism in MASLD, presenting potential new therapeutic targets. Oxidized phospholipids destabilize membranes and degrade into harmful byproducts like malondialdehyde (MDA) and 4-hydroxynonenal (HNE), which cross-link proteins and DNA, causing widespread damage, even at a distance from the site of production, and isoprostanes, prostaglandin-like compounds, which further exacerbate disease by promoting platelet adhesion to endothelial cells, highlighting platelet activation as another crucial factor in MASLD pathogenesis [27,28].

**Figure 3:**
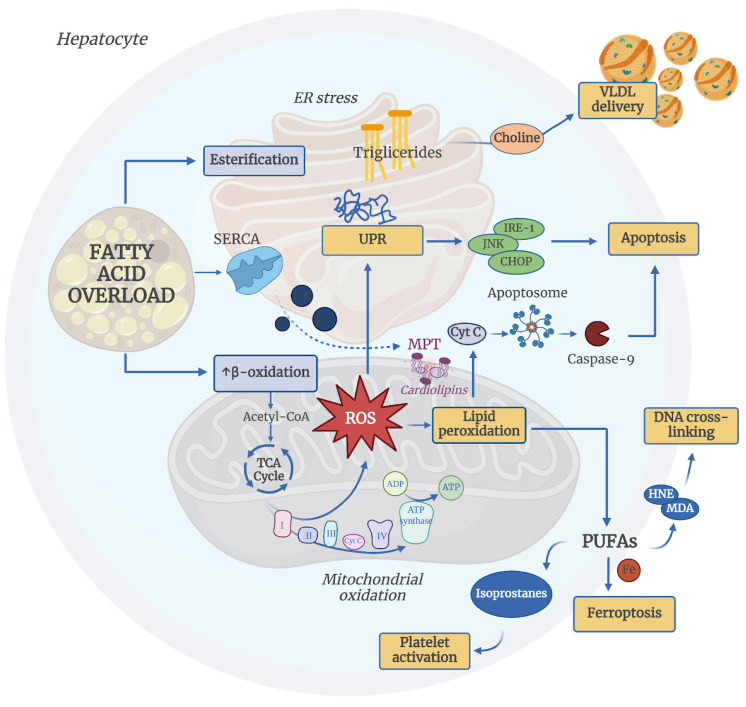
The role of oxidative stress in MASLD. ADP: adenosine diphosphate; ATP: adenosine triphosphate; Cyt C: cytochrome C; DNA: deoxyribonucleic acid; ER: endoplasmic reticulum; FE: ferrum; HNE: 4-hydroxynonenal; MDA: malondialdehyde; MPT: mitochondrial permeability transition; PUFAs: polyunsaturated fatty acids; ROS: reactive oxygen species; TCA: tricarboxylic acid; UPR: unfolded protein response; VLDL: very-low-density lipoproteins.

Oxidative stress is also responsible for the accumulation of misfolded and unfolded proteins in the lumen of the endoplasmic reticulum (ER), leading to the activation of the unfolded protein response (UPR) [29]. The UPR activates several downstream pathways, including IRE-1, JNK, and CHOP, which induce hepatocyte apoptosis [30]. Additionally, FFAs overload may decrease the activity of the SERCA pump, which transports Ca^2+^ into the ER. The calcium that leaks from the ER may act on mitochondrial membranes and open MPT pores [31], thereby promoting, as previously mentioned, cellular apoptosis. Figure 3 illustrates the involvement of oxidative stress in MASLD.

Damaged hepatocytes release DAMPs (Damage-Associated Molecular Patterns) into the extracellular space. Liver sinusoidal endothelial cells display scavenger functions, as they are equipped with high-affinity DAMP receptors. The recognition and subsequent internalization of DAMPs by these cells help to maintain immune homeostasis in the hepatic environment [32]. When these scavenger mechanisms become saturated, either due to an excess of DAMPs (as in the case of increased intestinal permeability) or because hepatic sinusoidal cells undergo capillarization (as seen in steatohepatitis and liver fibrosis), losing their scavenger function, DAMPs activate pattern recognition receptors (PRRs), such as Toll-like receptors (TLRs), on Kupffer cells (KCs) [32]. Activated KCs secrete TGF-β and PDGF, profibrogenic cytokines that directly stimulate hepatic stellate cells, mesenchymal cells responsible for extracellular matrix deposition and fibrosis development [33].

## 3. Role of Gut Microbiota in Hepatic Oxidative Stress and MASLD

### 3.1. Impairment of Intestinal Barrier and Bacterial Translocation

The intestinal barrier is a dynamic entity formed by different layers with the complex role of modulating nutrient absorption and pathogen rejection [34]. Starting from the lumen, the mucous layer hosts commensal microorganisms that form the gut microbiota. Mucus is composed of water (98%) and proteins called mucins, for the remaining percentage. MUC2 mucin, produced by goblet cells, represents the major component of this first luminal layer. Mucus exerts a double function: it acts as a physical spacer, protecting the epithelial layer from invasion by microorganisms, and is in fact renewed every 1–2 h. Secondly, MUC2 mucins form a large net-like structure that acts as a first size-exclusion filter [35].

Beneath this is the epithelial layer, where enterocytes are interconnected by junctional complexes. These include tight junctions (composed of occludin, claudins, and F-actin), adherens junctions (consisting of E-cadherin, α-catenin, and β-catenin), and desmosomes (which involve desmoglein, desmocollin, and desmoplakin). Beyond the epithelium lies the lamina propria, which contributes to immune defense through the secretion of immunoglobulin A (IgA), cytokines, and chemokines [36]. Aside from the epithelial barrier, a gut-vascular barrier (GVB) has also been described in the literature. GVB consists of endothelial vascular cells that control the type of antigens and particles that can translocate into the bloodstream [37]. A functional barrier allows two main pathways for molecules to cross into deeper layers: the “pore pathway”, a high-capacity, size- and charge-selective route, and the “leak pathway”, which is low-capacity and less selective. A third, “unrestricted pathway”, occurs in areas of epithelial damage, such as erosions and ulcers, allowing nonselective passage of luminal contents, including whole bacteria [37].

Disruption of the intestinal barrier, often caused by bacterial infections, oxidative stress, alcohol consumption, high-fat diets, and dysbiosis, leads to the condition commonly referred to as leaky gut syndrome, which exhibits a significant correlation with the “unrestricted pathway” [38]. This dysfunction has been linked to a variety of diseases, including celiac disease, inflammatory bowel disease, and MASLD [39,40,41,42,43]. In these scenarios, mucosal immune activation leads to increased IL-13 and TNF-α production. IL-13 upregulates claudin 2, increasing “pore pathway” permeability, whereas TNF-α enhances “unrestricted pathway” permeability by increasing myosin light chain kinase (MLCK) transcription and causing occludin endocytosis [44]. In MASLD, a link between gut dysbiosis, impaired intestinal barrier function, hepatic inflammation, and oxidative stress has been demonstrated in both animal and human models. Rahman et al. [42] reported that disruption of intestinal barrier integrity in mice fed a diet rich in saturated fats, fructose, and cholesterol exacerbated steatohepatitis. Similar findings were observed in humans. In 2019 our research group found that MASLD patients exhibited increased intestinal permeability, as evidenced by elevated urinary excretion of 51Cr-ethylene diamine tetraacetate (51Cr-EDTA), compared to healthy controls [43]. Additionally, the same study revealed decreased duodenal expression of zona occludens-1, a marker of tight junction integrity, in MASLD patients, underscoring the crucial role of increased intestinal permeability in the development of steatosis.

The disruption of intestinal barrier integrity permits bacterial endotoxins to enter the portal circulation and reach the liver. A key player in this process is lipopolysaccharide (LPS), a highly immunogenic molecule expressed on the surface of Gram-negative bacteria. Upon reaching the liver, LPS activates KC via sensing receptors, specifically TLR-4 receptors, triggering the release of TNF-α and TGF-β [45,46,47]. These cytokines, in turn, stimulate hepatic stellate cells to deposit extracellular matrix and promote the production of pro-inflammatory mediators, contributing to liver inflammation and fibrosis [48]. Studies have shown that mice lacking TLR4 exhibit resistance to both alcohol-induced liver damage and MASLD [49,50].

Additionally, endotoxins trigger lipid peroxidation in the liver, promoting the progression from simple steatosis to MASH and fibrosis [51]. Sakaguchi et al. observed a significant rise in hepatic lipoperoxide levels between 6 and 18 h following LPS exposure, which then gradually declined. In contrast, the liver activities of SOD and GPX, both key antioxidants, were reduced by 18 h post-endotoxin administration [51].

The gut microbiota is essential for maintaining intestinal barrier homeostasis and regulating gastrointestinal functions. It significantly influences the permeability and integrity of the gut barrier by supporting the repair and maintenance of tight junctions within the mucosal epithelium, protecting intestinal epithelial cells from damage caused by pathogenic bacteria [52]. For example, Bifidobacteria have been shown to reduce inflammation in different in vivo models, enhancing tight junctions through the stabilization of occludin and claudins 2 and 4, as well as exerting antioxidant properties, promoting IgA secretion, and stimulating maturation of immune cells in the gut epithelium [53].

*Lactobacillus royi* LR1 has demonstrated protective effects against damage caused by enterotoxigenic *E. coli*, primarily by preserving the zona occludens-1 and preventing its breakdown [54]. Additionally, this probiotic promotes the expression of tight junction proteins, contributing to the reinforcement of the intestinal barrier [55].

Short-chain fatty acids (SCFAs), such as butyrate, acetate, and propionate, which are produced by bacterial fermentation of indigestible fibers, have been shown to protect the gut barrier. Butyrate is primarily generated by Gram-positive Firmicutes, while acetate and propionate are predominantly produced by Gram-negative Bacteroides [56,57]. Butyrate, in particular, enhances MUC2 production [58], promoting mucin synthesis and improving the integrity of tight junctions by activating AMP-activated protein kinase (AMPK), which aids in the assembly of tight junction proteins [59].

### 3.2. Modifications in Bile Acid Structure

Bile acids (BAs), which are the primary components of bile, are classified into primary and secondary bile acids [60]. Primary bile acids (PBAs), such as cholic acid (CA) and chenodeoxycholic acid (CDCA), are synthesized from cholesterol in hepatocytes and then secreted into the bile duct. Secondary bile acids (SBAs), including lithocholic acid (LCA), deoxycholic acid (DCA), and ursodeoxycholic acid (UDCA), are derived from PBAs through bacterial metabolism in the small intestine [61]. Beyond their role in emulsifying dietary lipids, allowing their absorption, and in maintaining gut microbiota balance by inhibiting the growth of pathogenic bacteria [62], BAs interact with farnesoid X receptor (FXR) and G-protein-coupled bile acid receptor (TGR5) to regulate BA homeostasis, lipid metabolism, and glucose tolerance [63].

FXR is predominantly expressed on the surface of immune cells in the lamina propria of the ileum [63]. Upon activation, FXR exerts a negative feedback mechanism to suppress the activity of CYP7A1, the key rate-limiting hepatic enzyme involved in BA synthesis [64]. Additionally, FXR reduces the expression of SREBP-1c, which leads to a decrease in fatty acid and triglyceride synthesis in the liver, consequently mitigating hepatic steatogenesis and associated oxidative stress [65]. Notably, studies indicate that FXR activation can directly inhibit hepatic ferroptosis [66]. Moreover, FXR promotes hepatic glycogen synthesis by stimulating fibroblast growth factor 19 (FGF-19) [67]. Furthermore, activation of FXR by BAs down-regulates pro-fibrotic factors, such as transforming growth factor β1 (TGF-β1), tissue inhibitor of metalloproteinase 1 (TIMP1), and matrix metalloproteinase 2 (MMP-2), which promote hepatic stellate cells [68,69].

Moreover, activation of the TGR5 receptor on enteroendocrine L cells leads to the secretion of glucagon-like peptide-1 (GLP-1), which boosts insulin production, suppresses glucagon release from the pancreas, and reduces both appetite and gastrointestinal motility [70,71,72].

In MASLD patients, both plasma and hepatic BAs concentrations are increased [73,74], while both FXR and TGR5 pathways are downregulated, as demonstrated in animal and human studies [65,70,75,76]. This conflicting evidence can be attributed to the fact that not all BAs function as agonists for FXR and TGR5 receptors. In MASLD patients, alterations in the gut microbiota result in an overall increase in total BA levels, accompanied by a concurrent decrease in the proportion of BAs that act as FXR and TGR5 agonists. Zhang [77] and Cipriani [78] have shown that FXR knockout mice develop liver steatosis and hyperlipidemia, conditions that can be alleviated through FXR activation or overexpression. These findings suggest that alterations in the gut microbiota can influence BA composition and pool size, thereby disrupting down-signaling through FXR and TGR5.

In patients with MASLD, Jiao et al. observed elevated levels of both PBAs and SBAs in the serum. There was an increased abundance of Escherichia and Bilophila, bacteria known to metabolize taurine and glycine, leading to reduced levels of unconjugated BAs—those capable of crossing intestinal membranes and activating FXR [67]. Additionally, the proportion of deoxycholic acid (DCA), which acts as an FXR antagonist, was higher, while chenodeoxycholic acid (CDCA) levels were reduced [67]. Consistent with FXR downregulation, CYP7A1 gene expression was elevated, indicating increased BA synthesis in hepatocytes [67]. This BAs overload disrupts membrane integrity, activating cytosolic phospholipase A2 (PLA2) and releasing arachidonic acid, which is then metabolized by lipoxygenase (LOX) and cyclooxygenase (COX) to produce ROS [79,80].

Interestingly, Parseus et al. discovered that FXR knockout mice on a high-fat diet (HFD) did not develop steatosis, unlike wild-type mice on the same diet, and displayed a distinctly altered gut microbiota profile [81]. Furthermore, a recent study identified a novel BA, 3-succinylated cholic acid (3-sucCA), which was negatively associated with liver damage in MASLD patients. This BA supports the growth of *Akkermansia muciniphila* and is produced by *Bacteroides uniformis*, a strain found to be deficient in MASLD patients [82].

These findings highlight the need for more research in humans to better understand the complex interplay between gut microbiota, BAs, and the pathogenesis of MASLD/MASH.

### 3.3. Trimethylamine N-Oxide (TMAO) and Choline Metabolism

Growing evidence highlights the crucial role of the gut microbiota in the development of MASLD, particularly through the influence of metabolites such as trimethylamine N-oxide (TMAO) [83]. TMAO is naturally present in marine crustaceans, fish, meat, eggs, and soy, but can also be derived from dietary precursors such as choline, betaine, and L-carnitine. Choline metabolism primarily occurs in the large intestine, facilitated by the action of the gut microbiota [84]. Trimethylamine (TMA)-producing bacteria, which possess the genes encoding the choline-TMA lyase enzyme and its activating protein, metabolize dietary choline into TMA [85]. Once produced, TMA is absorbed and transported to the liver via the portal circulation, where it is metabolized into trimethylamine N-oxide (TMAO) by the hepatic enzyme flavin mono-oxygenase (FMO) [86]. Finally, TMAO is distributed to organs, where it can be eliminated (kidneys) and accumulated (tissues).

To note, TMA production is strongly influenced by the composition of an individual’s gut microbiota. Interestingly, less than 1% of intestinal microbes possess the genes required for TMA synthesis, yet even these low-abundance microorganisms are sufficient to drive significant TMA production [87]. This highlights the pivotal role of gut bacteria in this process.

Elevated levels of TMA and TMAO have been linked to increased activity of Firmicutes and Proteobacteria, both key TMA producers [86,87]. Additionally, a higher Firmicutes/Bacteroides ratio, characterized by a rise in Firmicutes and a reduction in Bacteroides [88,89], as Bacteroides lack the ability to produce TMA [90,91].

In a meta-analysis by Theofilis et al., TMAO levels were evaluated in a total of 7583 MASLD patients, demonstrating that patients with MASLD had significantly elevated circulating TMAO levels compared to individuals with a healthy liver [92]. More recent studies have shown that the TMAO levels not only contribute to the progression of MASLD but are also strongly associated with the disease’s severity and with the overall mortality in MASLD patients [93,94].

The mechanism underlying the harmful effects of TMAO on the liver is as follows: TMAO may disrupt carbohydrate, triglyceride, and cholesterol metabolism by inhibiting bile acid (BA)-mediated hepatic FXR signaling [95]. This suppression leads to a decrease in bile acid production by downregulating critical enzymes such as CYP1A1 and CYP27A1 and impairing bile acid enterohepatic circulation by inhibiting the organic anion transporter (OAT), which is essential for bile entry into hepatocytes [95]. The resulting impact on hepatic oxidative stress has been discussed previously. Evidence that TMAO-induced liver damage is mediated through its inhibitory effect on FXR signaling is supported by a study by Miyata et al., which found that in FXR-null mice, the exogenous administration of TMAO did not worsen MASLD but even improved it, as it reduced hepatic bile acid and cholesterol levels through a direct, FXR-independent mechanism [96].

Furthermore, recent findings indicate that TMAO compromises both the structure and function of the intestinal barrier in the colon, activating the TLR4/MyD88/NF-κB and downregulating the Wnt/β-catenin pathway [97]. This disruption increases the translocation of bacteria and LPS from the intestine into portal circulation, contributing to inflammation and liver oxidative stress [44,45].

Finally, TMAO contributes to endothelial dysfunction by suppressing nitric oxide (NO) synthesis, which results in the capillarization of liver sinusoidal endothelial cells and influences macrophage polarization [98]. These are just a few of the molecular mechanisms associated with the steatogenic effects of TMAO; however, emerging evidence suggests the involvement of additional molecular pathways, as demonstrated in vitro studies [99].

Given the harmful health effects associated with TMAO, a metabolite of choline, it may be erroneously assumed that reducing dietary choline intake could confer health benefits. However, because choline is an essential nutrient, diets low in choline can lead to changes in gut microbiota and various health issues. Gut bacteria convert TMA, which is then transformed into TMAO in the liver. This process reduces choline bioavailability and increases lipid accumulation in hepatocytes, potentially resulting in steatosis and even MASH [100,101,102]. Choline deficiency contributes to the development and progression of MASLD in several ways: First, choline is crucial for synthesizing phosphatidylcholine, a key component of VLDL. Without adequate choline, VLDL cannot be secreted and instead accumulates in hepatocytes as lipid droplets, leading to increased lipid peroxidation [103]. Additionally, a choline-deficient diet in mice downregulates the Wnt/β-catenin pathway, disrupting the GVB [37,104].

### 3.4. Promotion of Ferroptosis

Ferroptosis is a non-apoptotic cell death mechanism that involves free intracellular iron or iron-containing enzymes that react with oxygen and PUFAs to generate high levels of membrane lipid peroxides. The execution starts with membrane lipid peroxidation, forming highly reactive lipid radicals. This causes an aberrant movement of iron across the plasma membrane that eventually induces cell swelling and plasma membrane rupture. Overall, ferroptosis is a regulated process with a role in homeostasis that only occurs under unusual circumstances associated with disease, such as tumor suppression, or during para-physiological stimuli like acute or chronic inflammation [105].

Bacteria that compose the gut microbiome are crucial in the production of metabolites like SCFAs, bile acids, and neurotransmitters, which are implicated in the ferroptosis pathway. It has been seen that bacteria that act as probiotics can inhibit ferroptosis by chelating dietary iron, suppressing ROS production or accelerating their clearance, and downregulating key enzymatic reduction agents [106].

Probiotics, such as Bifidobacteria and Lactobacillus, promote iron absorption by forming essential amino acids or SCFAs to optimize dietary iron bioavailability [107]. Moreover, certain *E. coli* species have developed mechanisms to assimilate iron and remove substrates derived from the Fenton reaction, which would generate highly reactive hydroxyl radicals from H_2_O_2_ and Fe^2+^, avoiding potential inflammatory responses [108]. Nevertheless, other species, such as *Pseudomonas aeruginosa*, use bacterial lipoxygenases to trigger ferroptotic death in epithelial cells [109].

Also, diet can indirectly modulate ferroptosis: excessive oral iron supplementation can alter the microbiome composition, causing microenvironment alterations that put the basis for the development of different intestinal and systemic diseases, although more research is needed to better characterize the exact mechanisms of this complex interplay [110].

Studies show that iron overload is prevalent in patients with MASLD, insulin resistance, and obesity, suggesting that iron-induced lipid peroxidation, and thus ferroptosis, could be a trigger for dysmetabolic conditions [111]. Aberrant iron accumulation in the liver is able to initiate the Fenton reaction; this brings the production of ROS, which react with phospholipids of PUFAs, leading to the depletion of long-chain PUFAs in the cellular membrane. Long-chain PUFAs regulate lipid metabolism in the liver by repressing SREBP1c and promoting beta-oxidation by activating PPARα. Keeping this in mind, it appears plausible to speculate that the depletion of long-chain PUFAs in ferroptosis contributes to the pathogenesis of MASLD [112].

Serum ferritin is a clinical biomarker for detecting iron homeostasis in the body; its levels are an expression of iron stores and inflammation [113]. In a study involving 628 biopsy-proven MASLD adult patients, a 1.5-fold increase in serum ferritin levels above the normal limit was associated with higher steatosis grade, lobular inflammation, and advanced hepatic fibrosis [114]. Armandi et al. evaluated the relationship between baseline serum ferritin levels and longitudinal liver-related events in a cohort of 1342 patients with biopsy-proven MASLD from different centers. Over a median follow-up of 96 months, hyperferritinemia was associated with a 50% increased risk of liver-related events and 27% of all-cause mortality, suggesting the potential role of serum ferritin levels for predicting long-term prognosis of patients with MASLD [115].

Overall, the results of recent and ongoing research imply that ferroptosis may be a therapeutic target for MASLD/MASH treatment [116]. Different ferroptosis inhibitors, such as Fer1 and Tβ4, have been demonstrated in animal models to neutralize ROS and ameliorate the inflammatory damage of the liver [117,118]. Similarly, flavonoids like quercetin restore the balance of the gut microbiota and have been proven to inhibit ferroptosis in the liver [119]. Time-restricted feeding (TRF), defined as a regular calorie intake at indicated times, showed positive results in a MASH mouse model induced by a high-fat and high-fructose diet by downregulating genes that play a role in the ferroptosis cascade [120].

To our knowledge, therapeutic strategies involving ferroptosis have only been studied in animal and human cell line models. One of the reasons is that optimal conditions, such as limiting genetic and environmental factors and obtaining tissue samples, are easily applicable only in these settings. Furthermore, well-designed and large randomized controlled trials are needed to apply animal model findings to humans.

### 3.5. Platelets Activation

While there is substantial evidence supporting the role of leukocytes in maintaining the inflammatory response in MASLD, they are not the only cells involved. Recent studies on platelet hyperreactivity suggest that platelets play a significant role in the regulation of inflammatory and oxidative liver damage [28,121].

In response to endothelial damage within hepatic sinusoids, platelets adhere to von Willebrand factor (vWF) and subendothelial collagen via their glycoprotein receptors, GPIb-V-IX and GPVI [122]. Once activated, platelets undergo a conformational change in their GPIIb/IIIa receptor, enabling binding to fibrinogen and promoting platelet-platelet aggregation [122]. A study by our research group compared platelet behavior in 24 non-obese, non-diabetic patients with histology-confirmed MASLD/MASH with 17 healthy controls, revealing a greater presence of platelets and platelet aggregates in the liver sinusoids of MASH patients [123]. Another critical receptor, CD44, plays a role in the early and late stages of MASH pathogenesis by mediating platelet interactions directly with KCs [124,125]. This interaction, facilitated through platelet attachment to hyaluronic acid in the extracellular matrix via CD44, initiates an inflammatory and oxidative response linked to metabolic stress [126]. Studies using genetic and pharmacological inhibition of CD44 and hyaluronidase have shown reduced hepatic accumulation of platelets and KCs, which leads to decreased inflammation and an improvement in the MASLD activity score [127].

The gut microbiota can become a key driver of platelet activation, as demonstrated by several studies. Dysbiosis, for example, can compromise the intestinal barrier, allowing bacterial components like LPS to enter the systemic circulation, as previously discussed [52,53]. LPS, through its interaction with platelet TLR4, initiates downstream signaling pathways that enhance platelet granule secretion [128]. This cascade amplifies platelet activation and aggregation in response to common agonists, driven by the excessive production of eicosanoids like thromboxane A2 and F2-isoprostanes, as well as oxidative species generated by NOX2 [129]. LPS can also indirectly promote platelet aggregation by decreasing the activity of ADAMTS13, a key enzyme regulating platelet adhesion [130]. Furthermore, LPS-stimulated platelets interact with neutrophils, leading to the formation of neutrophil extracellular traps (NETs), which, in turn, expose tissue factor (TF) and activate the coagulation cascade [131,132]. NETs, in particular, have been shown to modulate the inflammatory environment in MASH by attracting monocyte-derived macrophages and may play a role in the progression from MASH to hepatocellular carcinoma (HCC) and in promoting metastasis [133].

Recent studies, though primarily focused on cardiovascular diseases rather than MASLD, have identified gut microbiota-derived metabolites that can directly activate platelets. For example, phenylacetylglutamine (PAGln), a metabolite produced by gut bacteria belonging to the Bacteroides, Firmicutes, and Proteobacteria phyla, is formed through a two-step process: dietary phenylalanine is converted into phenylacetic acid (PAA), which is then conjugated with glutamine by liver enzymes to produce PAGln [134,135]. PAGln is a prothrombotic compound that enhances the adhesion of P-selectin-positive platelets to the collagen matrix under physiological shear flow. This suggests that PAGln may drive platelet hyperreactivity, potentially contributing to platelet-mediated thrombosis and inflammation, thus exacerbating liver injury [136].

The gut microbiota also plays a role in the metabolism of trimethylamine N-oxide (TMAO), as previously discussed. TMAO activates platelets by promoting the release of intracellular calcium (Ca^2^⁺) stores. By producing inositol 1,4,5-trisphosphate (IP3) from membrane phospholipids, TMAO triggers the release of Ca^2^⁺ within platelets, resulting in platelet activation. Animal studies have shown that acute increases in circulating TMAO can enhance thrombosis potential in vivo [97,137].

It is highly likely that future molecular research will uncover additional gut microbiota-derived metabolites capable of promoting platelet aggregation, which could serve as therapeutic targets. One of the latest discoveries is 2-methylbutyrylcarnitine, which binds to integrin α2β1 on platelets, enhancing the activation of cytosolic phospholipase A2 (cPLA2) and promoting platelet hyperresponsiveness [138].

A limitation of the statements presented in this paragraph is that the correlation between gut-derived metabolites and platelet activation has not yet been investigated in MASLD-specific models. Therefore, additional studies are needed to determine whether these metabolites play a definitive role in the pathogenesis and progression of MASLD.

## 4. Factors Modulating Gut Microbiota in MASLD

### 4.1. Air Pollution

Air pollution is the second highest risk factor for noncommunicable diseases [139]. In 2019, 99% of the world’s population was living in places where the World Health Organization (WHO) recommended air quality levels were not met [140].

Air pollution consists of various substances, including gaseous toxic elements (e.g., carbon monoxide, nitrogen dioxides, sulfur dioxide, and ozone), as well as volatile organic compounds (e.g., benzene, toluene, ethylbenzene, acetone, and ethyl acetate) and particulate matter (PM). PM, which predominantly contributes to health issues related to air pollution, is divided into categories based on particle size: coarse particles (PM_10_, <10 μm), fine particles (PM_2.5_, <2.5 μm), and ultrafine particles (PM_0.1_, <1 nm). Coarse particles (PM_10_) are largely produced by industrial activities, while fine (PM_2.5_) and ultrafine particles (PM_0.1_) mainly originate from vehicle emissions [141]. Air pollutants can enter the gastrointestinal tract either through the mucociliary clearance of inhaled pollutants or by consuming contaminated food and water [142]. Animal studies have increasingly shown that exposure to ambient air pollution is linked to MASLD [143,144,145,146,147], although human epidemiological evidence is more limited (Table 1) [148,149]. One Chinese cross-sectional study involving 90,086 participants found that long-term exposure to ambient PM_0.1_, PM_2.5_, and PM_10_ is associated with an increased risk of MASLD. Specifically, for each 10 μg/m^3^ increase in PM_0.1_, PM_2.5_, and PM_10_, the odds ratios (ORs) for MASLD were 1.13 (95% CI 1.10–1.17), 1.29 (1.25–1.34), and 1.11 (1.09–1.14), respectively. These associations may be further intensified by unhealthy lifestyle choices and central obesity [149].

Two primary mechanisms may explain how air pollution contributes to the development of MASLD [150]. First, components of air pollutants may directly affect gut epithelial cells, leading to increased production of ROS and pro-inflammatory oxidative lipids, which can trigger intestinal and systemic inflammation. For example, some organic and metal PM components, such as quinones, can serve as the substrate of CYP450 to catalyze the generation of ROS directly, or polycyclic aromatic hydrocarbons, belonging to the subset of PM_2.5_, can be transformed into quinones by CYP450 and induce oxidative stress indirectly [151].

Furthermore, PM_0.1_ is particularly effective at depleting intracellular antioxidants like glutathione and stimulating ROS production and oxidative stress markers, such as heme oxygenase-1 (HO-1), in macrophages and bronchial epithelial cells [152]. Similarly, PM can cause ROS generation in intestinal epithelial cells, leading to disrupted intestinal tight junctions, increased gut permeability, and activation of the pro-inflammatory NFκB pathway [153]. Additionally, air pollution promotes the expression of genes involved in lipoperoxidation. For instance, exposure to diesel exhaust particles (a PM_2.5_ compound) has been shown to upregulate key genes in the 5-lipoxygenase pathway, such as arachidonate 5-lipoxygenase (Alox5) and glutathione peroxidase 6 (GPx6), resulting in significant increases in 5-HETE levels, a biomarker of lipid peroxidation, in both the liver and intestine [154].

Secondly, air pollutants impact the composition of the intestinal microbiome. Exposure to environmental pollution reduces both *alpha* and *beta* diversity of the human gut microbiota [155]. *Alpha* diversity refers to the variety of species within a single sample, while *beta* diversity reflects the variation in microbial communities between different samples [156]. Studies have shown that decreased *alpha* and *beta* diversity are linked to increased intestinal permeability. This is characterized by reduced expression of intestinal epithelial barrier proteins such as tight junction protein occludin and mucin 2 (Muc-2) in the duodenum and ileum, and increased expression of matrix metalloproteinase 9 (MMP-9), inflammatory markers like TNF-α and IL-1β, and TLR-4 [155]. Although findings can be inconsistent, there is evidence suggesting that air pollution affects the abundance of short-chain fatty acid (SCFA)-producing bacteria, decreases the presence of *Lactobacilli* and *Bifidobacteria*—both known for their anti-inflammatory properties—and reduces the relative abundance of *Actinobacteria*, which play a significant role in lipid metabolism, particularly accumulation and uptake [157]. Specifically, levels of *Actinobacteria* in the gut are inversely related to plasma LPC18:1, a metabolic marker associated with MASLD in severely obese individuals [158].

**Table 1:** Microbiome and non-microbiome changes induced by air pollution.

Environmental Intervention	Model	Microbiome Changes	Non-Microbiome Changes	Reference
Exposure to PM_2.5_ × 6 wk	Mice	Not studied	↑ IL-6 ↑ NAS score in liver sections ↑ Fibrosis in liver sections	[143]
Exposure to PM_2.5_ × 10 wk	Mice	Not studied	Insulin resistance ↓ PPARγ and PPARα Steatosis Hepatic inflammation Hepatic fibrosis	[144]
Exposure to PM_2.5_ × 10 wk	Mice	Not studied	Hepatic stellate cell activation (↑ α-SMA) ↑ TGFβ1 ↑ ROS	[145]
Residential air pollution exposure	Human	Not studied	↑ CK-18	[146]
Exposure to PM_2.5_ × 24 wk	Mice	Not studied	Insulin resistance Hyperlipidemia ↑ ALT, AST ↑ TNF-α, IL-1β, IL-18, IL-6 ↑ ROS	[147]
Cross-sectional study of >90,000 individuals exposed to high residence levels of air pollutants	Human	Not studied	Radiological steatosis Metabolic dysregulation	[149]
Gastrointestinal exposure to PM via gavage	Mice	Not studied	↑ ROS NF-κB activation Disruption of tight junctions ↑ Gut permeability	[153]
ApoE^−/−^ mice exposed to wood smoke or mixed diesel and gasoline engine emissions × 50 days	Mice	↑ Clostridioides ↑ Bacteroides ↓ Firmicutes ↓ Gut bacterial diversity	↑ MMP-9 ↓ MUC2	[155]

### 4.2. Physical Activity

Physical activity is widely recognized for its broad range of health benefits, including its ability to lower the risk of cardiovascular and gastrointestinal disease, obesity, diabetes, and mental health disorders [159,160]. Notably, exercise has been found to exert positive effects on hepatic steatosis, regardless of weight loss [161,162,163,164,165,166].

Multiple etiopathogenetic mechanisms are at play: both endurance and high-intensity exercise have been shown to reduce inflammation and enhance insulin secretion by upregulating glucagon-like peptide-1 (GLP-1) in the gastrointestinal tract and pancreas [165,166,167,168]. Aerobic exercise also increases adiponectin levels, a critical factor in mitigating inflammation associated with obesity and liver disease [169]. Additionally, physical activity promotes muscle growth, which triggers the release of interleukin-6 (IL-6), directly influencing glucose and lipid metabolism [170]. Moreover, physical activity appears to have the capacity to modulate the gut microbiota and decrease the risk of MASLD, though the underlying mechanisms remain unclear.

Numerous animal studies have described the effects of physical activity on gut microbiota composition (Table 2), whereas human studies remain limited. In a recent study by Clarke et al. [171], the impact of exercise on gut microbiota was examined in rugby players compared to two sedentary control groups: one with low BMI and the other with high BMI. The study found that athletes had significantly higher gut microbiota diversity than both control groups, which were matched for body size, age, and gender. The proportions of several gut microbial taxa also differed between athletes and controls, with minimal differences observed between the two sedentary groups. Specifically, the athletes exhibited a higher relative abundance of *Firmicutes* and *Proteobacteria*, along with a reduced abundance of *Bacteroides* [171]. Notably, *Akkermansia* was present in significantly greater proportions in both athletes and the low BMI group compared to the high BMI group [171]. *Akkermansia muciniphila*, a mucin-degrading bacterium residing in the mucus layer, has been shown to prevent the progression from simple steatosis to steatohepatitis by enhancing intestinal barrier function [172]. This is achieved through the reinforcement of intestinal tight junction proteins [173,174,175,176] and increased expression of short-chain fatty acids (SCFAs) [177]. Additionally, *Akkermansia muciniphila* suppresses liver inflammation by activating FXR on ileal immune cells and TLR2 on hepatic *gamma-delta* T cells, inducing CD4+ T cell anergy [178,179,180]. In line with these findings, the athletes in Clarke’s study exhibited lower inflammatory markers and improved metabolic indicators compared to both control groups, particularly the high BMI group, underscoring the athletes’ superior health profile [171].

While the exact mechanisms remain unclear and methodological variations complicate cross-study comparisons, current evidence suggests that exercise may influence the composition, diversity, and abundance of gut microbiota, thus preventing lipid peroxidation in patients with MASLD. Several potential pathways include the promotion of beneficial bacterial growth [181]; enhanced production of short-chain fatty acids (SCFAs) that aid in the repair of the intestinal barrier and support mucosal immunity [160,182,183,184]; modulation of bile acid composition and improvement of cholesterol turnover [185]; reduced blood flow to the gastrointestinal vessels, which limits the entry of endotoxins into systemic circulation [186]; and alterations in gut transit time, which affect substrate availability for the microbiota [187]. Further research is necessary to explore the effects of exercise on the gut microbiota and may help explain the differential response to exercise in MASLD patients.

**Table 2:** Microbiome and non-microbiome changes induced by physical activity.

Exercise Intervention	Model	Microbiome Changes	Non-Microbiome Changes	Reference
Male rugby athletes compared with normal BMI and overweight men	Human	More microbial diversity in athletes with ↑ Bacteroides ↑ Lactobacillus ↑ Akkermansia	Not studied	[171]
Forced treadmill running 40 min/day vs. voluntary wheel running × 6 wk	Mice	↑ Proteobacteria ↑ Tenericutes	Not studied	[181]
Forced treadmill running × 6 wk vs. sedentary controls	Mice	↓ Bifidobacteria ↑ *Clostridium leptum*	Not studied	[188]
Forced treadmill running 30 min/5 d/wk × 4 wk	Rat	↑ Allocaculum ↑ Pseudomonas ↑ Lactobacillus ↓ Streptococcus ↓ Aggregatibacter	Not studied	[189]
Forced wheel running 60 min/5 d/wk	Mice	↑ Firmicutes ↓ Streptococcus ↓ Bacteroides	Not studied	[190]
Peak effort in patients with Myalgic Encephalomyelitis/Chronic Fatigue Syndrome	Human	↑ Actinobacteria ↑ Firmicutes	Not studied	[191]
60 min/3 d/wk resistance exercise × 8 wk	Human	Not studied	Intrahepatic lipid reduction (15% to 12.2%)	[161]
60 min/3 d/wk of high intensity interval training (2 min of treadmill followed by 2 min rest) × 6 wk	Mice	Not studied	Statistically significant (*p* < 0.01) reduction in ALT and AST levels	[167]
20 min/3 d/wk of high-intensity intermittent exercise (8 s of cycle ergometer followed by 12 s rest) × 15 wk	Human	Not studied	Statistically significant reduction in fasting insulin levels	[169]
Concurrent aerobic and resistance exercise (metanalysis of 12 pediatric studies)	Human	Not studied	Statistically significant (*p* < 0.01) reduction in LDL levels	[170]
30 min/3 d/wk supervised cycle ergometer sessions × 4 wk	Human	Not studied	Intrahepatic triacylglycerol concentration ↓ 21% (8.55% to 6.79%)	[192]
45 min/6 d/wk with 60–70% estimated HRmax maintained for 20 min/session × 48 wk	Human	Not studied	From baseline: ALT ↓ 47%, AST ↓ 48%	[193]

### 4.3. Cigarette Smoke

Cigarette smoking is a key risk factor in the development and progression of MASLD [194,195,196]. Smoking contributes to MASLD through various mechanisms, including directly inducing oxidative stress in hepatocytes, altering lipid metabolism, damaging the intestinal barrier, and disrupting the gut microbiota (Table 3). Notably, cigarette smoke reduces antioxidant capacity (such as SOD activity) and increases inflammation in animal models [197,198]. It also increases the activity of HMG-CoA reductase, the rate-limiting enzyme in cholesterol synthesis [199], and triggers a dose-dependent activation of AMPKα1, which impacts lipid metabolism by promoting an increase in ceramide levels [200]. Ceramides are implicated in the progression of liver steatosis to steatohepatitis through their involvement in inflammation and fibrosis [201]. Cigarette smoke also affects adipose tissue by promoting lipolysis, leading to increased levels of circulating FFAs, which, as previously described, have significant effects on hepatocyte oxidative stress [202,203]. Additionally, although the precise molecular mechanisms remain unclear, cigarette smoking appears to impact the gut epithelium and mucin secretion by reducing prostaglandin E2 release and enhancing NO activity [204].

However, the primary focus of this section is the effect of cigarette smoking on the gut microbiota and its subsequent impact on hepatic oxidative stress. Thus, cigarette smoking is emerging as a cause of dysbiosis, and its effect varies also according to the section of the intestine involved. For example, Tomoda et al. described in rats how a 4-week exposure to cigarette smoke led to a reduction in acetic acid, propionic acid, butyric acid, and valeric acid in the cecum with a consequent increase in pH level and a simultaneous reduction in Bifidobacterium population [205]. Multiple studies have demonstrated that cigarette smoke exposure leads to a reduction in both alpha and beta diversity of the gut microbiota, with a decrease in Firmicutes and Proteobacteria and an increase in Bacteroides [200,205,206,207,208,209]. After smoking cessation, however, a reversal is observed, with increases in Firmicutes and Actinobacteria and a decrease in Bacteroides and Proteobacteria [210,211,212]. These shifts in microbial composition enhance the microbiota’s ability to extract energy from food. For instance, as demonstrated by Fluhr et al., fecal microbiota transplantation from smoke-exposed mice into germ-free mice resulted in significant weight gain across various diets and strains [213]. In the study by Jian Ge et al., cigarette smoke exposure significantly increased the abundance of Peptoclostridium, Turicibacter, and Clostridium, while Prevotella and Ruminococcaceae were significantly decreased [200]. These findings suggest that the alterations in lipid metabolism induced by cigarette smoke exposure are closely linked to disruptions in the gut microbial community. Moreover, the study revealed that rats exposed to cigarette smoke exhibited decreased activity of the enzyme CYP7A1, the rate-limiting step in bile acid synthesis, along with lower fecal concentrations of SCFAs and increased TMAO activity. These findings suggest that smoke-induced dysbiosis influences various molecular pathways, though the precise mechanisms behind this etiopathogenic connection are still not well understood [200]. Furthermore, it remains unclear whether a correlation exists between the extent of dysbiosis and the level of cigarette smoke exposure. Among the studies referenced, only Nolan-Kenney et al. investigated this relationship, revealing a dose-dependent increase in pro-inflammatory bacteria linked to xenobiotic metabolism, particularly the genera Slackia and Collinsella, often found in individuals with a dyslipidemic phenotype [211]. Further studies are necessary to confirm these data.

Several studies have shown that smoking cessation can also be a risk factor for MASLD. This may be attributed to the weight gain often observed in individuals who have recently quit smoking. The gut microbiota is involved in this process as well. Nicotine, the primary component of cigarettes, affects the nervous system by suppressing appetite and regulating body weight [214]. A study on mice explored this phenomenon, suggesting that dysbiosis and changes in smoking-related metabolites may create a feedback loop that influences the anorexia typical of active smoking. Upon cessation, the anorexic signaling quickly fades, but the obesogenic microbiome associated with smoking is eliminated more gradually, leading to weight gain [213].

**Table 3:** Microbiome and non-microbiome changes induced by cigarette smoke.

Environment/Intervention	Model Used	Non Microbiome Changes	Microbiome Changes	Reference
Exposition to cigarette smoking for 4 weeks	Rats	↑ pH	↓ Bifidobacterium	[205]
Current, former, and never smokers	Human	Not studied	↑ Bacteroides ↓ Firmicutes ↓ Proteobacteria	[215]
Benzo[a]pyrene oral exposure	Mice	Not studied	↑ Bacteroides ↑ Paraprevotella ↑ Alcaligenaceae ↓ Lactobacillus ↓ Oscillospira	[216]
Benzene exposure	Mice	Not studied	↑ Helicobacter	[217]
Smokers and non-smokers	Human	Not studied	↑ Firmicutes ↓ Bacterioides ↑ Actinobacteria ↓ Proteobacteria	[210]
Tobacco smokers, electronic cigarette smokers, and non-smokers	Human	Not studied	↓ Bacteroides ↑ Prevotella	[218]
Cigarette smoking	Human	Not studied	↑ *B. thetaiotaomicron* ↑ *B. massiliensis* ↑ *L. amylovorus*	[219]
Cigarette smokers	Human	Not studied	↑ Bacteroides ↑ Actinomyces ↓ Firmicutes	[220]
Healthy tobacco smokers and healthy non-smoker	Human	Not studied	↑ *B. vulgatus* ↓ Firmicutes ↓ Actinobacteria	[221]
Tobacco smokers	Human	Not studied	↑ Firmicutes ↑ Actinobacteria ↑ Proteobacteria	[211]
Cigarette smoking	Human	Not studied	↑ Desulfovibrio	[222]
Never, former, and current smokers	Human	Not studied	↑ Prevotella ↓ Lachnospira ↓ Tenericutes ↑ Veillonellaceae	[223]
Volunteers selected by pre-test and pre-questionnaire	Human	Not studied	↑ Bacteroides	[224]
Electronic cigarette, tobacco smokers, and controls	Human	Not studied	↑ Prevotella ↓ Bacteroides	[212]
Smokers and non-smokers	Human	Not studied	↓ Enterococcus	[225]
Nicotine by subcutaneous pomp	Rats	Not studied	↑ Actinobacteria ↓ Firmicutes	[226]
Exposure to formaldehyde	Mice	Not studied	↑ Actinobacteria ↑ Proteobacteria ↓ Cyanobacteria	[227]
Exposure to arsenic	Mice	Not studied	↓ Firmicutes	[228]
Cigarette smoking	Human	Not studied	↑ Prevotella ↓ Bacteroides	[229]
Cigarette smoking exposure	Mice	↑ Cholesterol liver content ↑ Cholesterol synthesis genes expression ↑ Insulin resistance	↑ Verrucomicrobia ↑ Epsilonbacteraeota ↓ Firmicutes ↓ Actinobacteria ↓ Patescibacteria	[230]
Cigarette smoking exposure	Mice	Not studied	↑ Bacteroides ↑ Prevotellaceae	[231]
Current, previous, and never smokers	Human	Not studied	↑ Firmicutes ↓ Bacteroides ↓ Actinobacteria ↓ Prevotella ↓ Neisseria	[232]
Cigarette smoking exposure	Mice	↑ IL-6	↑ Lachnospiraceae	[233]

### 4.4. Alcohol

Alcohol consumption disrupts gut homeostasis through its direct effects on both the gut microbiota and the intestinal epithelium, creating a compounded impact [234]. Ethanol-induced alterations in the gut microbiota lead to dysbiosis and bacterial overgrowth, changing the distribution of bacterial species such as Bacteroides, Firmicutes, and Proteobacteria (Table 4). This alteration has been extensively studied in both mice and humans.

Understanding the molecular alterations induced by alcohol in mice has been pivotal in advancing research on alcohol-related liver diseases (ALD). Building on the Lieber-DeCarli diet—a balanced liquid diet providing a portion of total caloric intake from alcohol (ethanol), initially used in ALD patients to investigate alcohol’s metabolic and pathological effects—researchers have developed various alcohol exposure regimens to better humanize the murine model [235].

In mice, chronic alcohol exposure reduces bacterial diversity, decreasing Bacteroides and Firmicutes while increasing Proteobacteria and Actinobacteria [236]. In humans, similar microbial shifts occur, with an increase in Proteobacteria, considered potential biomarkers of dysbiosis due to their pro-inflammatory properties [237,238], and a decrease in Actinobacteria and Firmicutes [239,240]. Alcohol is particularly associated with a relative increase in endotoxin-producing Enterobacteriaceae (a family within the Proteobacteria phylum) and a reduction in taxa that produce SCFAs, such as Lachnospiraceae and Ruminococcaceae (both belonging to the Firmicutes phylum) [241,242]. Supporting this, the analysis of the fecal metabolome in humans with alcohol use disorders reveals a reduction in SCFAs [243].

These microbial alterations compromise gut functions, for example, hindering nutrient digestion (i.e., reducing intestinal motility) [244], and worsening immune regulation and pathogen defense [245,246]. Alcohol exacerbates this dysfunction by increasing the permeability of intestinal epithelial cell membranes [247,248]. In rodent studies, alcohol-induced changes in intestinal permeability have been associated with alterations in the gut microbiota, particularly through decreased activity and expression of hypoxia-inducible factor 1α (HIF-1α). These effects were reversed by treatment with the probiotic *L. rhamnosus GG*. HIF-1α is also crucial in the development of alcohol-related hepatic steatosis [249]. Wang et al. demonstrated that alcohol reduces the expression of mRNA involved in synthesizing tight junction proteins in a Caco-2 cell model, further weakening the intestinal barrier [250]. Additionally, acetaldehyde, a toxic metabolite of alcohol, directly disrupts tight and adherens junctions in the colonic epithelium, further contributing to gut barrier breakdown [251].

As a result of this compromised barrier, LPS can translocate into the portal circulation, where they interact with TLR4, triggering signaling pathways that include KC activation and NADPH oxidase activation, leading to ROS production. This cascade promotes liver inflammation and injury [252,253], consistent with findings of increased plasma antibodies against LPS in patients with steatohepatitis [254]. Early alcohol exposure also initiates gut leukocytosis and the release of ROS, leukotrienes, and histamine from mast cells, with matrix metalloproteinase-9 (MMP-9), IL-6, and TNF-α contributing to inflammation and increased gut permeability [255].

Collectively, these effects lead to structural changes in the intestine (such as increased wall thickness, villus elongation, and crypt depth), contributing to oxidative stress linked to gut inflammation, which drives the progression of metabolic dysfunction-associated alcohol-related liver disease (MetALD) [256,257].

Finally, alcohol consumption alters gut microbiota diversity, which indirectly leads to changes in BA composition. This results in an increased proportion of SBAs, a higher total bile acid concentration, and a shift towards glycine-conjugated BAs instead of taurine-conjugated ones [234,258]. This effect is believed to be due to dysbiosis reducing taurine availability and an enhanced rate of enterohepatic cycling [16,17]. Although the full implications of these changes remain unclear, it is likely that glycine-conjugated BAs (which become more prevalent with alcohol intake) and continued bile acid synthesis (despite high luminal concentrations) contribute to the development of hepatic lipidic overload [234].

**Table 4:** Microbiome and non-microbiome changes induced by alcohol consumption.

Environment/Intervention	Model Used	Non Microbiome Changes	Microbiome Changes	Reference
Alcohol-exposed (5% *v*/*v*) group and alcohol non-exposed group for 6 weeks	Mice	Tight junction disruption ↑ Endotoxemia ↑ Fecal pH ↑ Hepatic steatosis ↑ Hepatic inflammation ↑ Hepatic injury	↓ Bacteroides ↓ Firmicutes ↑ Proteobacteria ↑ Actinobacteria	[236]
Alcohol overconsumption for >10 years and no or low alcohol intake	Human	↓ Butyric acid ↓ Muscle mass ↓ Vitamin C plasma level	↑ Proteobacteria ↑ Sutterella ↑ Clostridium ↑ Holdemania ↓ Faecalibacterium ↓ Actinobacteria	[239]
Alcoholic liver disease group and healthy subjects	Human	↑ Endotoxemia	↓ Bacteroides ↑ Proteobacteria	[240]
Alcohol solution 10% 24 h/d for three months	Rats	Tight junction disruption ↑ Endotoxemia	↑ Ruminococcus ↑ Coprococcus ↑ Streptococcus	[259]
Alcohol or isocaloric diet for 3 weeks	Mice	↑ Endotoxemia ↓ c-type lectins Reg3b ↓ Reg3g	↑ Verrucomicrobia ↑ Bacteroides ↓ Firmicutes	[260]
Alcohol consumption for at least 6 months and non alcoholic healthy control	Human	↑ Endotoxemia	↑ Fusobacteria ↑ Actinobacteria ↑ Firmicutes ↓ Bacteroides	[261]
Alcohol chronic consumption and non-alcoholic volunteers	Human	Not studied	↑ Streptococcus ↓ Bacteroides ↓ Bifidobacterium ↓ Ruminococcus	[262]
Alcohol use disorder patients and low-risk drinking subjects	Human	↑ Endotoxemia ↑ TNF-α ↑ IL-1β	↑ Bacteroides ↓ Akkermansia	[263]
Comparison among moderate and severe alcoholic hepatitis, heavy drinking controls, and healthy controls	Human	Not studied	↑ Firmicutes ↑ Proteobacteria ↓ Bacteroides	[264]
Alcoholic hepatitis	Human	Not studied	↑ Veillonella ↓ Bacteroides ↓ Akkermansia	[265]
Alcohol consumption	Human	Not studied	↑ Bacteroides ↓ Firmicutes	[220]
Alcohol consumption	Human	Not studied	↑ Acidaminococcus ↑ Bacteroides	[225]
Alcohol moderate consumption in MASLD patients	Human	Not studied	↑ Bacteroides ↑ Bifidobacteriaceae ↑ Streptococcaceae ↑ Ruminococcaceae	[266]

### 4.5. Diets

The impact of diet on human health is well-established, with much of its influence being mediated through changes in the gut microbiota (Table 5). The microbiota is shaped not only by the type and number of macronutrients consumed but also by the timing of food intake. For instance, fasting can increase luminal pH, change the composition of the mucus layer, and impact the habitat of the gut microorganisms [267,268].

There are several types of diets, with the most emblematic being the Western diet and the Mediterranean Diet. The Western diet is particularly rich in saturated fats, refined carbohydrates, and simple sugars (such as fructose and sucrose), but low in fruits and vegetables, and it represents a significant risk factor in the pathogenesis of MASLD [269]. Most studies investigating the correlation between diet and MASLD have been conducted in animal models, with the human Western diet being reflected in the murine high-fat diet (HFD), consisting of about 60% fat, 20% protein, and 20% carbohydrate [270], or high-fat high-sugar diet (HFHSD), consisting of about 13.1% proteins, 60.6% lipids, and 26.3% carbohydrates [271].

Chronic overconsumption of sugars, fats, and proteins has been associated with a rise in Bacteroides spp. and *Ruminococcus torques* [272,273]. In mice subjected to an HFD, Velazquez et al. reported an increase in Firmicutes and Actinobacteria, accompanied by a decrease in Bacteroides and Tenericutes [274]. Furthermore, molecular markers of ER stress in these mice revealed heightened activity of the JNK and CHOP pathways [274]. The exact mechanism linking these gut microbiota shifts to liver stress is still under investigation, but intestinal permeability changes due to dysbiosis could be a contributing factor. In particular, sulfate-reducing bacteria (SRB) are found in higher amounts in mice consuming HFD [275,276]. Sulfide, produced in excess by SRB like *Bilophila wadsworthia*, can break down disulfide bonds within the mucus by disrupting the polymeric MUC2 network, a key element in maintaining the integrity of the mucus layer and supporting mucosal repair [271]. Research suggests that diets high in saturated fats promote SRB growth, resulting in a compromised mucus barrier, heightened gut inflammation, and increased microbial translocation [271]. Additionally, a study by Ming Yang et al. demonstrated that mice on a Western diet showed an elevated presence of *Blautia producta*, a bacterium capable of producing 2-oleoylglycerol (2-OG), a metabolite that crosses the intestinal barrier, enters the enterohepatic circulation, and activates hepatic stellate cells to generate extracellular matrix in a macrophage-dependent manner [277]. This observation was further validated by the detection of 2-OG accumulation in liver biopsies of patients with MASH [277].

On the other hand, the Mediterranean Diet, characterized by high levels of fiber and unsaturated fatty acids along with low amounts of saturated fats and simple sugars, has demonstrated protective effects against MASLD [278,279,280]. The fiber and vegetables in this diet help prevent the overgrowth of potentially pathogenic bacteria, such as *E. coli* and other Enterobacteriaceae [281]. The fermentation of non-digestible fibers by the gut microbiota generates SCFAs, such as butyrate, propionate, and acetate [282]. These SCFAs play a crucial role in supporting the intestinal epithelium, acting as the primary energy source for enterocytes and maintaining the integrity of the gut barrier [283]. Research has also shown that omega-3 polyunsaturated fatty acids (PUFAs) can help rebalance the gut microbiota by restoring the Firmicutes/Bacteroides ratio and promoting the growth of Lachnospiraceae species, which are known to boost the production of the anti-inflammatory SCFA butyrate [284,285,286].

Furthermore, recent studies have highlighted the potential benefits of intermittent fasting on MASLD, with the gut microbiota emerging as a key mediator of these effects. Periods of fasting appear to induce significant shifts in the microbiota, which in turn influence metabolic processes. In a rodent model, intermittent fasting was found to enhance SCFA production through microbial fermentation [287]. Additionally, the fasting regimen promoted a microbiota-dependent shift from white to brown adipose tissue, reducing fat accumulation in liver cells. Indeed, brown adipose tissue is known for its role in thermogenesis, and by upregulating UCP1, it stimulates lipolysis and glycogenolysis, contributing to fat consumption [288]. Another study reported that intermittent fasting in HFD-fed rodents increased Bacteroides levels, reduced Firmicutes, and lowered liver triglyceride content and hepatic steatosis. Furthermore, a randomized clinical trial in healthy adult males showed that intermittent fasting not only improved serum lipid profiles and liver function but also enhanced microbial diversity, particularly boosting Prevotellaceae and Bacteroidaceae populations [289]. While these findings point to the positive effects of fasting on gut microbiota and liver health in animal models, more human research is needed to fully understand the impact of various fasting regimens on MASLD through gut microbial modulation.

**Table 5:** Microbiome and non-microbiome changes induced by diet.

Type of Diet	Model	Microbiome Changes	Non-Microbiome Changes	Reference
HFD	Mice	↑ *Bacteroides* spp. ↑ Ruminococcus torques ↑ *E. coli*	↓ SCFAs	[272]
Western diet	Human	↑ *Bacteroides* spp. ↓ Prevotella	Not studied	[273]
HFD	Mice	↑ Firmicutes, Ruminococcus ↓ Turicibacter, Anaeroplasma	ER stress	[274]
HFHSD	Mice	↑ Bilophila wadsworthia	Inflammation, susceptibility to colitis	[271]
HFD	Mice	↑ Blautia producta	Liver inflammation, hepatic steatosis	[277]
HFHSD	Mice	↑ Dorea ↑ Oscillospira ↑ Ruminococcus	Promote dysbiosis and insulin resistance	[290]
Mediterranean Diet	Human	↑ Bifidobacterium ↑ Roseburia ↑ Lactobacillus	↓ Pro-inflammatory arachidonic acid levels	[284]
	Human	↓ Faecalibacterium ↓ *Akkermansia* spp.	Not studied	[285]
	Human	↑ Lachnospiraceae	Not studied	[286]
	Human	↑ Prevotella ↓ Bifidobacteria	Beneficial changes in body weight; Cardiometabolic impairment	[291]
	Human	↓ Actinobacteria ↑ Bacteroides	Protective effects against inflammation and dysbiosis	[292]
	Human	↑ Agathobaculum ↑ Anaerostipes	Not studied	[293]
Intermittent fasting	Mice	↑ Bacteroides ↑ Prevotella ↓ Firmicutes	Protective effects against obesity and hepatic steatosis; ↓ Liver triglycerides	[289]
	Human	↑ Bifidobacterium ↑ Lactobacillus	Lipid profile improvement	[294]
	Human	↑ Ruminococcus ↑ Roseburia ↑ Clostridium	↑ SCFA ↓ LPS	[295]
	Human	↑ Bacteroides	Not studied	[296]
	Human	↑ Bacteroides ↑ Firmicutes	↑ Serum levels of butyrate	[297]

HFD: high-fat diet; HFHSD: high-fat/high-sugar diet.

## 5. Discussion

Oxidative stress plays a pivotal role in the pathogenesis and progression of MASLD, serving as a key mediator in the complex interplay between metabolic dysfunction, inflammation, and liver injury. Central to this process is the excessive production of ROS, driven by hepatic lipid overload, which in turn induces mitochondrial dysfunction, ER stress, and chain-reaction lipid peroxidation, creating a pro-oxidative environment that overwhelms the liver’s antioxidant defenses, ultimately leading to cellular damage, apoptosis, and fibrogenesis [10].

The various pathogenic mechanisms contributing to the “multiple parallel hits” hypothesis in MASLD, which converge on increased hepatic oxidative stress, are directly or indirectly influenced by individual gut microbiota composition [52,53]. Primarily, alterations in gut microbiota composition weaken the intestinal barrier’s resistance to stressors, leading to increased intestinal permeability [52,53]. This facilitates the translocation of endotoxins, including LPS produced by Gram-negative bacteria, into the portal circulation. LPS, through TLR4 activation, stimulates KCs and hepatic stellate cells, promoting chronic low-grade hepatic inflammation and fibrogenesis [45,46,48]. Additionally, dysbiosis alters bile acid composition and subsequently disrupts enterohepatic circulation. In MASLD, there is a prevalence of bile acids that antagonize intestinal receptors, such as TGR5 and FXR [78]. Downregulation of TGR5 signaling reduces GLP-1 production, resulting in decreased satiety, increased insulin resistance, and obesity, which are critical factors in the onset and progression of MASLD [70,71,72]. Furthermore, downregulation of the FXR signaling pathway disrupts cholesterol metabolism, reducing the mobilization of lipids from hepatocytes to other tissues [64,65,66]. Lipid accumulation in hepatocytes triggers mechanisms of iron-mediated programmed cell death, a process known as ferroptosis [111]. Notably, ferroptosis can be directly regulated by gut microbiota, as certain bacterial strains can modify systemic iron bioavailability [107]. Additionally, a small percentage of intestinal bacterial strains synthesize the enzyme TMA-lyase, which converts dietary metabolites such as choline, betaine, and L-carnitine into TMA, subsequently metabolized to TMAO in the liver [86]. TMAO not only compromises the integrity of intestinal barrier tight junctions and downregulates the FXR signaling pathway but also induces endothelial dysfunction in hepatic sinusoids [95,97,98]. Long-term endothelial dysfunction drives sinusoidal capillarization, a precursor to hepatic parenchymal fibrosis [98]. Furthermore, TMAO decreases ADAMTS13 levels, stimulating platelet aggregation and activation within the sinusoids [130]. Activated platelets release secretory granules and induce NET formation, which releases pro-inflammatory cytokines and oxidative enzymes [133]. Recent research has highlighted that certain gut microbiota-derived metabolites, such as phenylacetylglutamine and 2-methylbutyrylcarnitine, can directly activate platelets [136,138].

It is well known that environmental and lifestyle-related factors, such as air pollution, physical inactivity, cigarette smoking, alcohol consumption, and the Western diet, are risk factors in the pathogenesis of MASLD. These risk factors amplify oxidative stress in the liver both through direct mechanisms and indirectly by altering the composition of the microbiota [155,171,200,236,274]. In fact, all the aforementioned risk factors reduce the alpha and beta diversity of the gut microbiota, increasing intestinal barrier permeability. Low alpha and beta diversity are markers of an unhealthy microbiota and are associated with a decreased production of the protective mucus layer that lines the intestinal lumen, reduced expression of tight junction proteins, and increased production of enzymes such as metalloproteinases and of pro-inflammatory cytokines [156].

Moreover, these risk factors reduce the expression of bacterial strains capable of producing SCFAs, which are essential for ensuring the turnover and repair capacity of the intestinal barrier [158,177,200,234,241,242,272]. Often, these risk factors have a synergistic effect on dysbiosis and the promotion of MASLD. For example, alcohol intake alters the microbiota composition in a way that disrupts bile acid structure, impairing the activation of the FXR signaling pathway [214], while cigarette smoking reduces the expression of CYP7A1, the rate-limiting enzyme in bile acid synthesis [171]. Conversely, physical activity enhances FXR activation [178].

In summary, the intricate relationship between gut microbiota, oxidative stress, and the pathogenesis of MASLD underscores the need for a deeper understanding of these interconnected systems. While advances have been made in elucidating the pathways through which microbiota influence liver health, it is important to acknowledge the limitations associated with translating animal models to human clinical contexts, especially regarding gut microbiota research. Animal studies often utilize specific strains or controlled environments that do not accurately replicate the complexity and diversity of the human microbiome. Furthermore, variations in genetic backgrounds, dietary habits, and environmental exposures can yield different outcomes in humans compared to animal models. As noted, dysbiosis may manifest differently across species, and the interaction between gut microbiota and host metabolism can be influenced by numerous factors, including the presence of concurrent diseases or conditions unique to human physiology. These discrepancies underscore the need for caution when drawing parallels between animal models and human disease. While animal studies can provide valuable mechanistic insights, they must be complemented with rigorous human studies to validate findings and develop effective therapeutic strategies.

Furthermore, many alterations observed in microbiota composition often reflect the consequences rather than the causes of metabolic diseases, including MASLD. This nuance is critical, as it emphasizes that while specific bacterial strains may be identified as altered in disease states, their exact role in pathogenesis remains unclear. Moreover, the identification of certain bacterial species associated with MASLD does not necessarily imply a direct pathogenic role; rather, it highlights the complexity of microbial interactions and their contributions to disease. This complexity complicates the pursuit of targeted microbiota modulation therapies, necessitating further investigation to establish causal relationships. Approaches such as longitudinal studies, controlled interventions, fecal microbiota transplantation, and the use of germ-free or microbiota-humanized animal models are critical to disentangling causation from correlation. Interventions aimed at restoring a balanced microbiome could yield beneficial effects, not only by directly influencing microbial populations but also by addressing behavioral and environmental factors that negatively affect gut health. Lifestyle changes, dietary modifications, and the use of probiotics represent promising avenues to enhance gut microbiota diversity and resilience, potentially mitigating oxidative stress and liver damage associated with MASLD.

## Data Availability

No new data were created or analyzed in this study. Data sharing is not applicable to this article.
